# Surgical treatment of acromioclavicular joint dislocation of Rockwood III/IV: a retrospective study on clavicular hook plate versus arthroscopic TightRope loop titanium button

**DOI:** 10.1186/s12891-024-07269-5

**Published:** 2024-02-26

**Authors:** Yafei Wang, Chengzhen Ren, Junqi Niu, Le Cao, Can Yang, Fanggang Bi, Ke Tian

**Affiliations:** Department of Orthopedic Surgery, The First Affiliated Hospital of Zhengzhou University, NO.1 Jianshe East Road, Zhengzhou, 450052 China

**Keywords:** Arthroscopy, TightRope loop titanium button, Clavicular hook plate, Acromioclavicular joint dislocation

## Abstract

**Purpose:**

To compare the clinical efficacy of arthroscopic TightRope loop titanium button and clavicular hook plate in the treatment of acromioclavicular joint (ACJ) dislocation of Rockwood III/IV.

**Methods:**

A retrospective analysis of patients with ACJ dislocation in our hospital from January 2018 to December 2020 was conducted. The patients were assigned to be treated with arthroscopic TightRope loop titanium button (TR group) or clavicular hook plate (HP group). The preoperative, intraoperative and postoperative data and imaging findings of the two groups were compared.

**Results:**

A total of 58 eligible patients were enrolled in this study. Compared with HP group, TR group had shorter incision length and less blood loss during operation. Postoperative follow-up ranged from 12 to 24 months (mean 15.4 months). At 6 months and 12months postoperatively, compared with HP group, TR group had lower VAS and higher CMS, and the difference was statistically significant. At 12 months postoperatively, compared with HP group, TR group had lower ACJ gap and coracoclavicular joint(CCJ) distance, and the difference was statistically significant.In HP group, there were 3 cases of subacromial impact, 1 case of redislocation, 2 cases of traumatic arthritis and 2 cases of wound infection. There was 1 case of redislocation in TR group.

**Conclusions:**

Compared with clavicular hook plate, arthroscopic TightRope loop titanium button is minimally invasive, safe and effective in the treatment of ACJ dislocation, and has a good trend in clinical application.

## Introduction

 Acromioclavicular joint (ACJ) dislocation usually occurs in young and active people, accounting for 12% of shoulder injuries [1]. ACJ dislocation usually occurs between the lateral end of the clavicle and the medial end of the acromion [2]. Indirect or direct injury leads to rupture or avulsion of acromioclavicular ligament and coracoclavicular ligament, resulting in ACJ dislocation. ACJ injuries are 5 times more common in men and often involve contact sports [3].

The treatment of ACJ dislocation is usually guided by Rockwood classification. According to radiological criteria, Rockwood classification system classifies ACJ dislocation into Rockwood I-VI [4]. According to the guidelines, conservative treatment is generally recommended for Rockwood I and II, and surgical treatment for Rockwood IV and VI. However, the treatment of Rockwood III is still controversial [5, 6]. For Rockwood III, conservative treatment can be chosen for patients with less physical activity, basic diseases or complications, but surgical treatment is the first choice for professional athletes or sports enthusiasts. But usually, surgical treatment is the first choice for Rockwood type III and above injuries [7].

There are many methods for the treatment of ACJ dislocation, but there is no unified standard. For injuries above Rockwood III, the common methods are hook plate method, Kirschner needle tension band method, steel wire method, PDS sling method and Weaver-Dunn method [8]. In the past, clavicular hook plate internal fixation was the first choice for the treatment of acute acromioclavicular joint dislocation, which can provide anatomical reduction and rigid fixation of ACJ. However, secondary surgery is needed to remove the internal fixation in order to avoid pain during activity [9]. With the development of sports medicine, new materials such as adjustable suspension titanium button (TightRope, TightLoop) [10], Endobutton [11] fixation and arthroscopy-assisted treatment [12] of ACJ dislocation have emerged. Different from clavicular hook plate internal fixation, TightRope technique provides reduction of ACJ with small incision, which not only does not damage the surface of acromioclavicular joint, but also does not need a second operation to remove the implant and reduce the occurrence of complications.

Related clinical experimental studies evaluate and compare different surgical methods for the treatment of ACJ dislocation [13], but few studies analyze the clinical difference between clavicular hook plate internal fixation and arthroscopic TightRope technique in the treatment of ACJ dislocation. In 2018, we began to treat ACJ dislocation with TightRope loop titanium button under arthroscopy. Therefore, we conducted a retrospective study to compare the efficacy of arthroscopic TightRope loop plate and clavicular hook plate in the treatment of Rockwood III/IV ACJ dislocation. The purpose of this study was to compare and analyze the clinical surgical results, postoperative functional recovery and imaging findings of the two methods in the treatment of Rockwood III/IV ACJ dislocation.

## Methods

### Patients

Retrospective analysis of surgical data of ACJ dislocation treated in orthopaedics department of our hospital from January 2018 to December 2020. The inclusion criteria are as follows: 1. Rockwood type III or higher dislocation; 2. unilateral acute ACJ dislocation; 3. Any age, gender, or race. The exclusion criteria are as follows: 1. Patients with clavicle fracture, coracoid process fracture and vascular nerve injury; 2. Patients with combined osteoporosis; 3. Patients with combined severe heart, liver, lung and kidney insufficiency and infectious diseases. All patients knew their operation methods before operation and signed the operation informed consent form. According to the different fixation methods, they were divided into two groups. The HP group was fixed with clavicular hook plate technology, and the TR group was fixed with TightRope loop titanium button under arthroscopy. This study was approved by the Ethics Committee of the First affiliated Hospital of Zhengzhou University (No:2021-KY-1021-002), and all data were taken from medical records and X-rays. We hypothesize that there were differences in clinical operation, postoperative function and imaging findings between clavicular hook plate and TightRope loop titanium button under arthroscopy, and the TightRope technology can achieve good clinical and radiological effects.

### Surgical methods

#### Reduction of clavicular hook plate

**Fig. 1:**
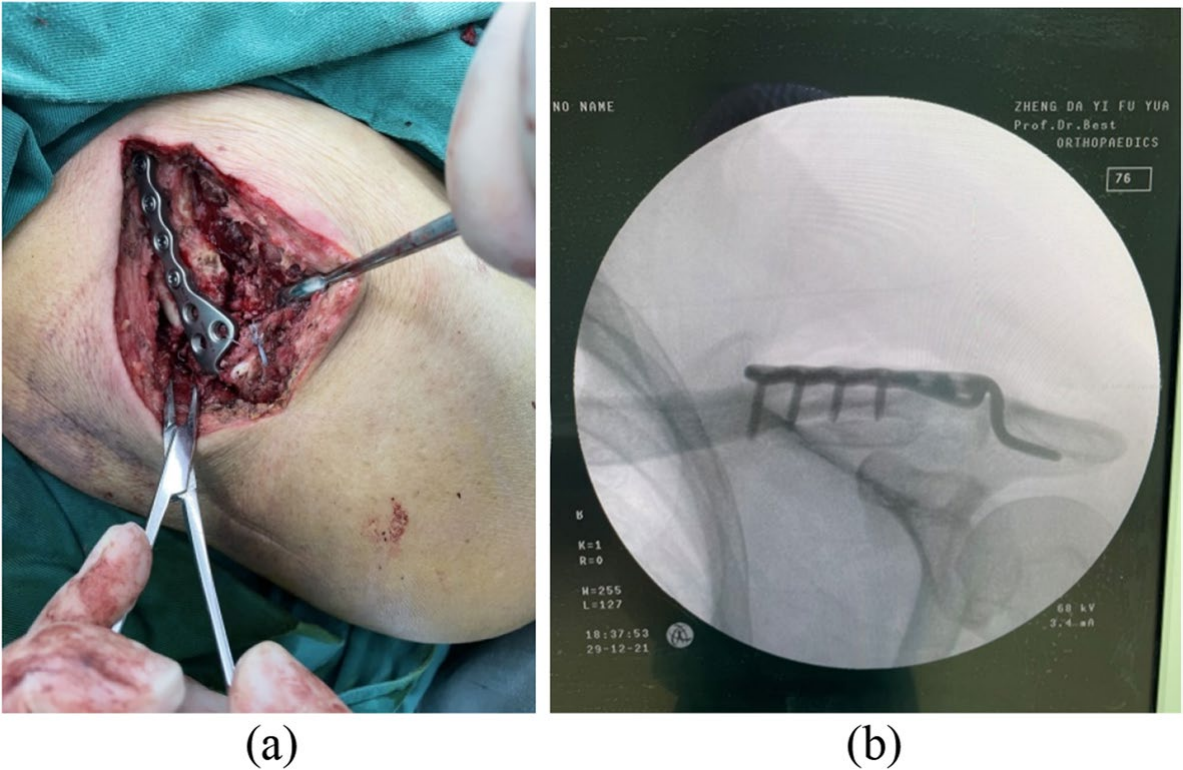
**a**: Dislocation of the ACJ with skin incision at all levels, exposure of the ACJ, and implantation of the clavicle hook plate, which can be seen as a surgical incision about 10 cm long; **b**: The C-arm X-ray device showed that the reduction of ACJ was good

All the operations are performed under general anesthesia and by a single surgeon. After satisfactory general anesthesia, the patient took the beach chair, the shoulder cushion was high, disinfected and covered with towel. A 10 cm incision was made at the center of the dislocation, and each skin layer was cut in turn. Exploring the ACJ, the acromioclavicular ligament was ruptured and the clavicular bone shifted upward. The ACJ was reduced, the clavicular hook locking plate was inserted, and four screws were screwed in for fixation. C-arm X-ray device showed that the reduction and fixation was good, and the movement of the affected clavicle was good. Hemostasis was sufficient, closed the incision layer by layer, and finished bandaging. A typical case is shown in Fig. 1.

#### Arthroscopic reduction of TightRope loop titanium button

**Fig. 2:**
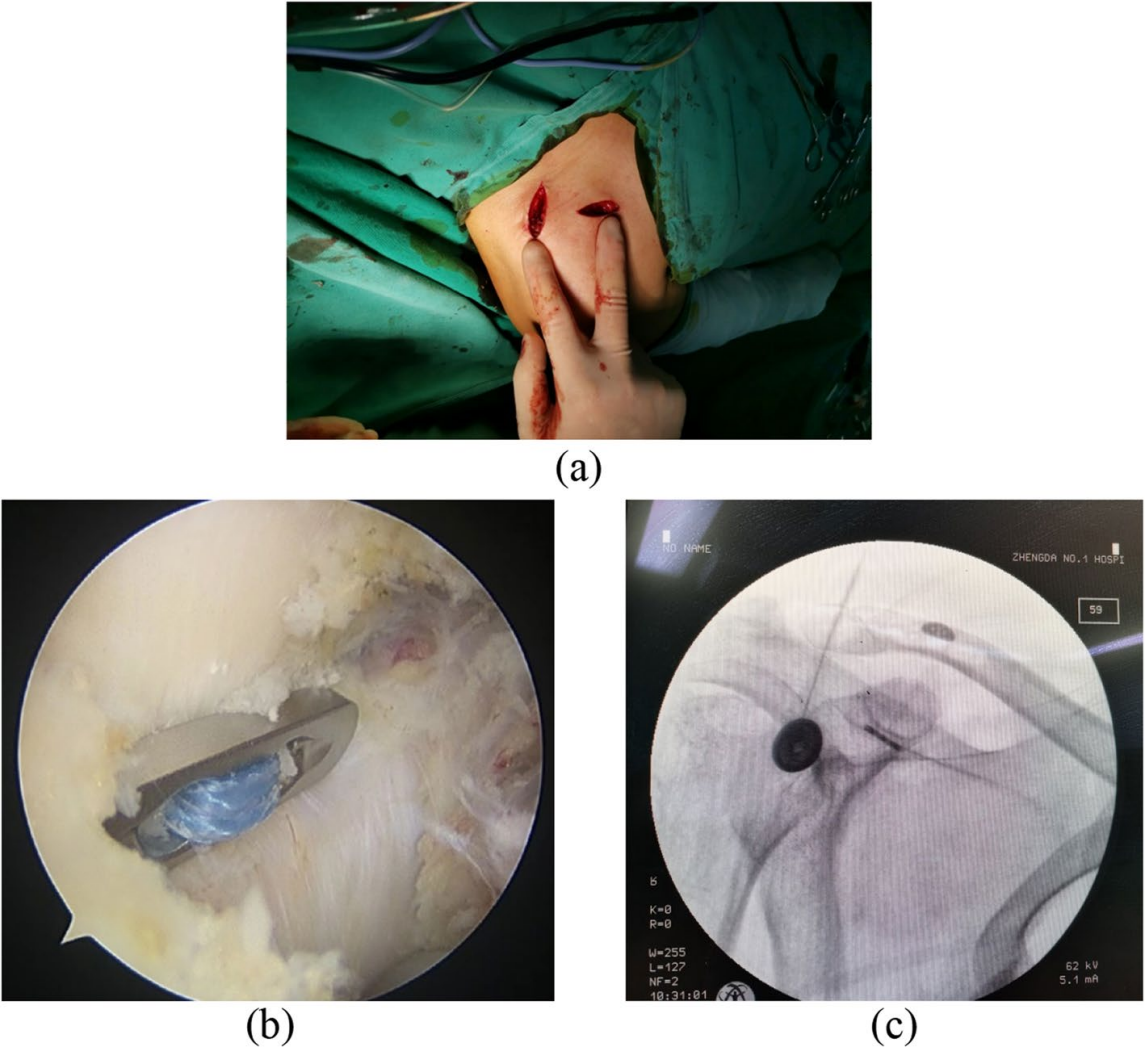
**a**: Arthroscopic surgical incision; **b**: the suspended titanium plate at the base of the coronoid process was observed under the arthroscope; **c**: the C-arm X-ray device showed good repositioning of the ACJ

After general anesthesia was satisfied, the patient took the beach chair, disinfected and covered with towel. The posterior lateral approach was taken, and the arthroscope and access pipe were inserted to explore the shoulder joint in turn. There was no obvious injury of articular cartilage, no obvious hyperplasia of synovium and no obvious inflammation of biceps long head. The anterolateral approach of the right shoulder was taken to establish a lateral channel and radiofrequency ablation was performed to reveal the base of the right coracoid process. A 2 cm incision was made centered on the medial side of the ACJ to expose the superior clavicular surface. The posterior cruciate ligament reconstruction locator was placed at the base of the coracoid process, and a Kirschner wire was inserted into the lateral 1/3 of the clavicle to drill the 4.5 mm bone tract. Used guide wire traction to place one side of the Tightrope suspension plate at the base of the coracoid process, tighten the suspension line and made the other side of the suspension plate close to the clavicle. C-arm X-ray device showed good reduction of ACJ. The incision was sutured and the dressing was completed .A typical case is shown in Fig. 2.

### Postoperative management

The patient was suspended by shoulder and neck wrist sling for 4 weeks after operation. Patients were encouraged to strengthen the movement of wrist and elbow joint 3 days after operation. Passive shoulder exercise was performed step by step at 3 weeks after operation. The sling was removed at 4 weeks after operation and active shoulder exercise was carried out step by step. The exercise of climbing the wall with fingers and touching the top of the head with hands were carried out step by step at 6 weeks after operation, so that the function of shoulder joint gradually returned to normal. Hot compress the affected shoulder for 10 min before doing all rehabilitation activities, and then cold compress for 10 min after rehabilitation exercise. The normal motor function of shoulder joint recovered gradually 3 ~ 4 months after operation.Hot compress the joints for 5 min before doing functional exercise, and then cold compress for 10 min after exercise.

### Observational indices

The operation-related indexes (incision length, operation time and intraoperative blood loss) and hospital stay were compared between the two groups; incidence of complications (subacromial impingement, traumatic arthritis, incision infection, redislocation).The X-ray examination of the affected shoulder was performed to measure the ACJ gap and coracoclavicular joint (CCJ) distance. Evaluations of shoulder function by Visual Analogue Scale (VAS) and Constant-Mueley Score (CMS).

### Statistical analysis

Sample size calculation: referring to the results of related literature [14], the postoperative CMS in HP group and TR group were 83.3 ± 8.8 score and 89.3 ± 4.2 score respectively. Assuming that α = 0.05, 1-β = 0.90, the 16 patients in each group should be included respectively by PASS15.0 software (NCSS, LLC, Utah, USA).

Analysis: Data analysis was performed using SPSS 21.0 statistical software (IBM Corp., Armonk, NY, USA). First of all, the measurement data use *Shapiro-Wilk* test to determine whether the data is of normal distribution. Measures are expressed as means ± standard deviations. Independent sample *t*-test was used for comparison between groups. Repeated measurement analysis of variance was used to compare intra-group and inter-group different time points. The counting data were tested by *χ*^*2*^*-*test. The difference was statistically significant (*P* < 0.05).

## Results

A total of 58 eligible patients were enrolled in this study, including 35 patients in the HP group and 28 patients in the TR group. The sample size was sufficient. All data are in accordance with normal distribution. The general data of the two groups were not statistically significant and can be compared (Table 1). All the operations were completed successfully, and no serious complications such as vascular, nerve damage and fracture occurred during the operation. The 58 cases were followed up for 12 ~ 24 months (mean 15.4months). The TP group was superior to the HP group in surgical incision and intraoperative blood loss. The shoulder function score of TR group was better than that of HP group after 6 months operation. In the HP group, there were 3 cases of subacromial impingement, 1 case of redislocation, 2 case of traumatic arthritis and 2 cases of incision infection. There was 1 case of redislocation in TR group, without wound infection. The complication rate in the TR group was much lower than that in the hooked plate group.

**Table 1:** General data and comparison of two groups of patients before operation *(*Mean ± SD*)*

	HP group (*n* = 35)	TR group (*n* = 23)	P
Mean age in years	45.86 ± 11.01	40.57 ± 11.42	0.083
Gender(male/female)	27/8	13/10	0.097
Course of disease (Day)	4.91 ± 1.67	5.00 ± 1.60	0.846
Dislocation side (Left/ right)	21/14	14/9	0.947
Rockwood typing (III/IV)	18/17	12/11	0.956

### The general information and comparison of patients between the two groups before operation

There was no significant difference in age, gender, injury side, injury time and classification between the two groups (*P*>0.05). Most of the patients in this study are young adults, with an average age of 46.3 years. And the sample was mainly male (68.97%, 40/58), there are twice as many male patients as female patients. And about three times as many male patients as female patients in the clavicular HP group, which is similar to the previous Mark [3] study. The patients were treated with clavicular hook plate or TightRope loop titanium button under arthroscopy at 4.9 days after injury.

### Evaluation of operation-related indexes and comparison of hospitalization time between the two groups

The incision length and intraoperative blood loss in the TR group were less than those in the HP group, and there was significant difference between the two groups (*P*<0.05). However, there was no significant difference in operation time and postoperative hospital stay between the two groups (*P*>0.05)( Table 2).

**Table 2:** Comparison of operation-related indexes and hospitalization time between the two groups *(*Mean ± SD*)*

	HP group (*n* = 35)	TR group (*n* = 23)	P
Incision length(cm)	7.74 ± 1.95	4.00 ± 1.65	< 0.001
Operation time(min)^a^	46.69 ± 3.78	48.30 ± 4.39	0.140
Intraoperative bleeding loss(ml)	61.43 ± 56.92	22.83 ± 20.38	0.001
Postoperative hospital stay(d)	7.69 ± 3.46	6.78 ± 3.12	0.317
Costs(yuan)^b^	10981.94 ± 341.61	13863.43 ± 1741.11	< 0.001

^a^Operation time refers to the time from incision to suture

^b^Costs refers to operation treatment cost and operation cost

### Results of VAS and CMS in both groups

**Fig. 3:**
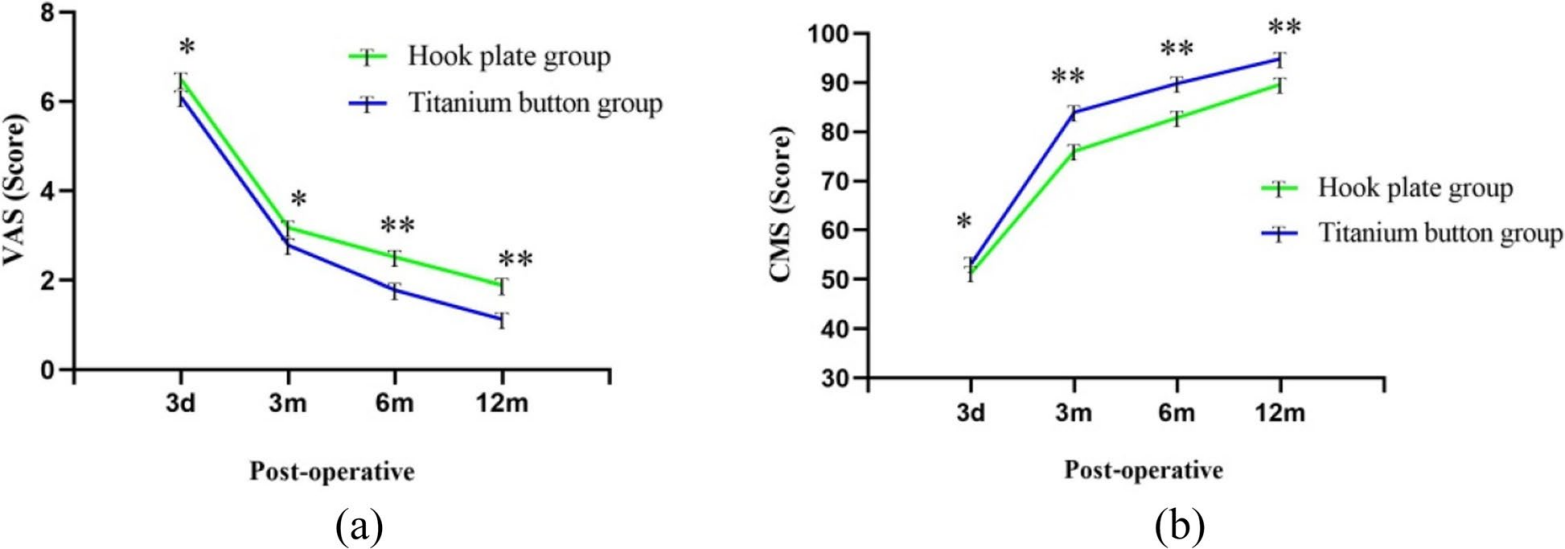
The VAS (**a**) and CMS (**b**) in HP group and TR group were compared. With the observation time, The VAS decreased and CMS increased in both groups. There were significant differences in VAS and CMS between the two groups at 6 months and 12 months after operation. *P>0.05, **P<0.05

With the passage of time, the VAS of the two groups decreased significantly, while the CMS increased significantly (Fig. 3), and there were significant differences at different time points after operation (*P* < 0.001). There was no significant difference in VAS and CMS between the two groups before operation (*P*>0.05). Three months after operation, the VAS of TR group was better than that of HP group, but there was no statistical significance (*P*>0.05). Six months after operation, the VAS of TR group was lower than that of HP group, and the difference was statistically significant (*P*<0.05). At 3 and 6 months after operation, the CMS of TR group was better than that of HP group, and the difference was statistically significant (*P*<0.001)( Table 3). The above data show that the patients treated with titanium button have less pain and better functional recovery than those treated with hook plate.

**Table 3:** Comparison of VAS and CMS between the two groups of patients preoperatively and 12 months postoperatively *(*Mean ± SD*)*

	Follow-up time	HP group (*n* = 35)	TR group (*n* = 23)	P
VAS	Preoperative	6.31 ± 1.28	6.00 ± 1.29	0.342
	Postoperative 3 days	6.49 ± 0.98	6.09 ± 1.04	0.145
	Postoperative 3 months	3.17 ± 1.07	2.78 ± 1.38	0.233
	Postoperative 6 months	2.51 ± 0.82	1.78 ± 1.09	0.005
	Postoperative 12 months	1.89 ± 0.87	1.13 ± 1.14	0.006
	*P*	< 0.001	< 0.001	
CMS	Preoperative	41.69 ± 11.85	41.00 ± 8.11	0.795
	Postoperative 3 days	51.31 ± 11.94	53.17 ± 6.53	0.498
	Postoperative 3 months	76.06 ± 3.73	82.87 ± 4.80	< 0.001
	Postoperative 6 months	82.86 ± 3.99	89.83 ± 3.86	< 0.001
	Postoperative 12 months	89.69 ± 2.71	94.83 ± 1.97	< 0.001
	*P*	< 0.001	< 0.001	

### Imaging measurements of patients in both groups

**Fig. 4:**
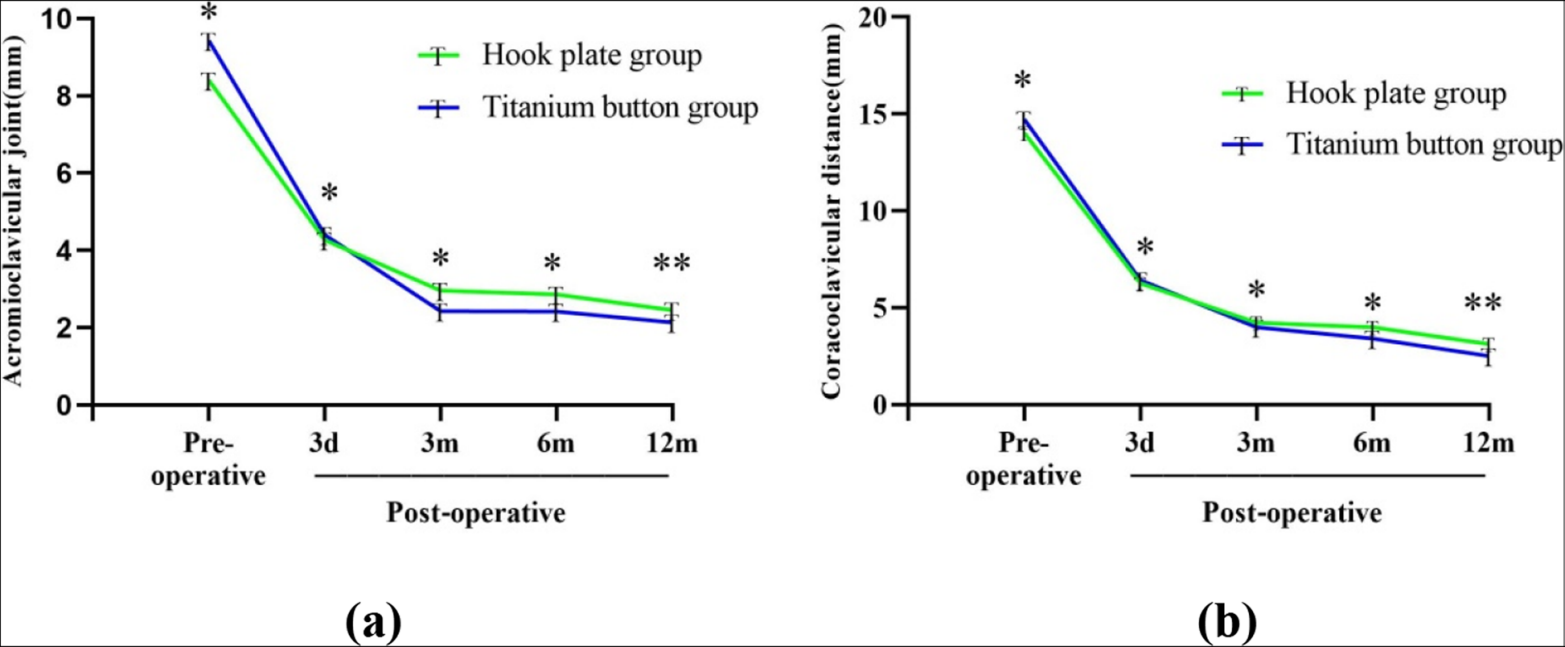
The ACJ gap (**a**) and CCJ distance (**b**) in HP group and TR group were compared. With the observation time, The ACJ gap and CCJ distance increased in both groups, There were significant differences between the two groups at 12 months after operation. *P>0.05, **P<0.05

**Fig. 5:**
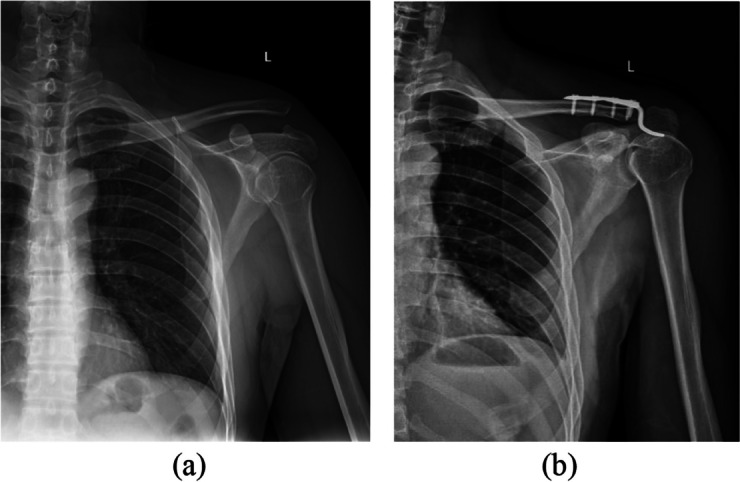
Basic information: male, left ACJ dislocation,(Rockwood type III), treatment with clavicular hook plate technique. **a** the preoperative positive X-ray film showed that the distal clavicle was raised and the space between ACJ and CCJ was widened; **b** the postoperative positive X-ray film showed that the reduction of the left ACJ was good

**Fig. 6:**
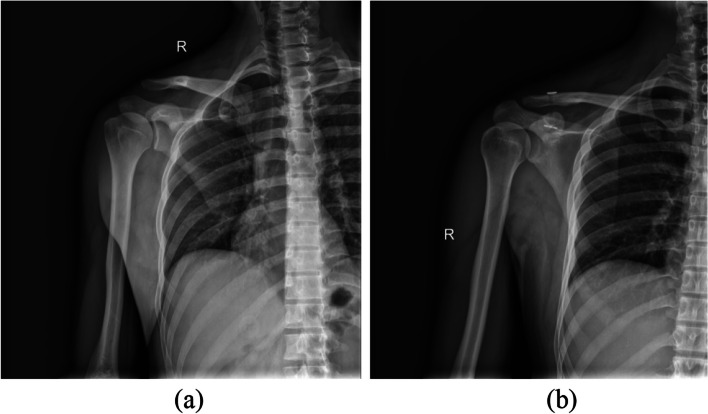
Basic information: male, right ACJ dislocation, (Rockwood type III) ,arthroscopic treatment with TightRope loop plate technique. **a**: the preoperative positive X-ray film showed elevation of distal clavicle and widening of ACJ gap and CCJ distance; **b**: the postoperative positive X-ray film showed good reduction of right ACJ

Compared with the preoperative period, the ACJ gap and CCJ distance were significantly reduced in both groups at different observation time points after surgery (Table 4; Fig. 4). There was no significant difference in ACJ gap and CCJ distance between the two groups before operation and 3 and 6 months after operation (*P*>0.05). By the time of the last follow-up, the internal fixation was in place in both groups without loosening or breaking, all patients returned to the normal range of motion and were very satisfied with the function of their shoulder joint. The images of two groups of typical cases are shown in Figs. 5 and 6.

**Table 4:** Comparison of imaging measurement results before and 12 months after operation between the two groups *(*Mean ± SD*)*

	Follow-up time	HP group (*n* = 35)	TR group (*n* = 23)	P
ACJ gap (mm)	Preoperative	8.40 ± 2.61	9.43 ± 3.16	0.185
	Postoperative 3 days	4.27 ± 1.51	4.40 ± 1.19	0.726
	Postoperative 3 months	2.96 ± 1.27	2.43 ± 1.24	0.122
	Postoperative 6 months	2.86 ± 0.91	2.42 ± 0.89	0.075
	Postoperative 12 months	2.45 ± 0.49	2.14 ± 0.43	0.018
	*P*	< 0.001	< 0.001	
CCJ distance (mm)	Preoperative	14.03 ± 4.12	14.72 ± 3.44	0.507
	Postoperative 3 days	6.26 ± 2.55	6.43 ± 3.06	0.818
	Postoperative 3 months	4.22 ± 2.09	3.99 ± 2.29	0.696
	Postoperative 6 months	3.99 ± 1.42	3.40 ± 1.20	0.107
	Postoperative 12 months	3.12 ± 0.78	2.50 ± 0.58	0.002
	*P*	< 0.001	< 0.001	

### Comparison of incidence of complications between the two groups

Compared with 22.86% of the HP group, 4.35% of the TR group had a significantly lower incidence of complications, but the difference was not statistically significant (Table 5).

**Table 5:** Comparison of incidence of complications between the two groups

	Hook plate group (*n* = 35)	Titanium plate group (*n* = 23)
Subacromial impingement	3	0
Redislocation	1	1
Traumatic arthritis	2	0
Incision infection	2	0
Total	8	1
Incidence of complications(%)	22.86	4.35
*P*	0.125	

## Discussion

In this study, compared with the HP group, the TR group had shorter incision, less intraoperative blood loss and fewer postoperative complications in terms of clinical results. The VAS and CMS were better than those in the HP group in terms of postoperative functional rehabilitation. The ACJ gap and CCJ distance in the HP group were lower than those in the HP group in terms of imaging findings. This study demonstrated that anatomic fixation and reduction of ACJ with the TightRope loop titanium button under arthroscopy can achieve good functional prognosis and stable radiological results.

The purpose of surgical treatment of ACJ dislocation is to reduce and fix the ACJ, repair the acromioclavicular ligament, coracoclavicular ligament and joint capsule, and restore the function of the ACJ. At present, clavicular hook plate is one of the most commonly used methods for the treatment of ACJ dislocation in clinical operation [15]. Its advantage is that joint reduction can be realized on both vertical and horizontal planes [16]. However, the studies have shown that postoperative complications such as subacromial impingement, subacromial osteolysis, shoulder pain, redislocation, rotator cuff injury and infection may occur [17–21]. Chen et al [22] through the treatment of patients with ACJ dislocation with clavicular hook plate, the functional indexes of shoulder joint were observed before and after treatment. Although his study concluded that this method of surgical treatment was effective, the plate must be removed within a certain period of time after operation. In this study, postoperative complications occurred in both groups, but the incidence of postoperative complications in TR group was significantly lower than that in HP group. One case of redislocation in the TR group was also caused by the high activity of the postoperative shoulder joint. Due to the small incision and the absence of large plate insertion, subacromial impingement and secondary surgery caused by plate insertion were avoided.

Qi et al [23] conducted a systematic review and Meta-analysis of TR and HP technology, the TR operation time ranges from 36.2 to 75 min, and the HP operation time ranges from 33.5 to 58 min. And they found that there was no significant difference in the time of different surgical procedures, which was consistent with the observation of this study (TR: 48.30 ± 4.39 min, HP: 46.69 ± 3.78 min).

Yu et al [24] included 112 patients with ACJ dislocation in the experimental study. The average age of 60 patients with HP fixation was 56.43 ± 9.64 years old, and the average age of 52 patients with TR was 50.15 ± 15.39 years old. There was no significant difference in age between the two groups. In the Meta-analysis conducted by Pan [25], the maximum age of patients with ACJ dislocation was 49.2 ± 16.9 years old, and the minimum age was 32.3 years old. In this study, the mean age of patients with ACJ dislocation was 46.3 years, which was within the range of their analysis.

The TightRope loop titanium plate technique (ARTHREX, USA) is a minimally invasive method for stabilizing the ACJ and strengthening the CCJ complex with high strength sutures. Cai et al [26] have shown that TightRope technique was superior to clavicular hook plate in the treatment of ACJ dislocation in terms of incision length, surgical blood loss, postoperative pain and avoiding secondary operation. In this study, the incision length and intraoperative blood loss in the TR group were better than those in the HP group. The reason is that the TR group reduces the damage to blood vessels and tissue during the operation, while the HP group needs to expose the clavicle and coracoid process in a large area during the operation, the partial release of the stop point of the deltoid muscle and extensive soft tissue peeling will lead to a large amount of bleeding during the operation and affect the postoperative recovery of the patients.

The studies have shown that TightRope loop titanium button can achieve good clinical and radiological results [2, 23]. In this study, we pay more attention to the evaluation of postoperative function of patients. After surgery, the VAS was significantly decreased and CMS was significantly increased in both groups, and the scores in the TR group were better than those in the HP group. This difference is due to the implantation of clavicular hook plate during operation, which leads to aggravation of shoulder pain and decrease of range of motion. And most of the patients need to remove the clavicular hook plate 6 months after operation, the second operation will further affect the movement of the shoulder joint. During the follow-up of this study, there were 3 patients with subacromial impingement in HP group, and the shoulder function was significantly improved after the steel plate was removed, which indicated that clavicular hook plate was closely related to shoulder dysfunction. However, the TR group avoided this kind of injury [27]. There was significant difference in ACJ gap and CCJ distance between the two groups at 12months after operation (*P<*0.05).The reason is that the titanium plate group mainly repairs and suspends the coracoclavicular ligament, which can bear the same or even greater tensile force than the original ligament in mechanics [28]. Surgical treatment should focus on stabilization of both vertical and horizontal components to improve the clinical outcome [29]. As a result, the stability of the TP group was better than that of HP group in horizontal plane and vertical plane after operation.Therefore, it will be a trend to treat ACJ dislocation with TightRope loop titanium button under arthroscopy [30].

Klemens et al [31] found that there was no significant difference in the cost of diagnosis, laboratory and imaging examination, aftercare and physical therapy when comparing TightRope techniques with other surgical techniques (K-wire fixation) in the treatment of ACJ dislocation. The main cost difference was the high material cost of TightRope technology. In this study, the cost of TR group was also higher than that of HP group. Although the TightRope technology itself was more expensive, the technology appears superior due to its ease of application, stable postoperative fixation, no need to remove the implant, avoidance of secondary surgery, and lower total hospital costs.

According to the above studies, in the process of surgical treatment of patients, TightRope technology can achieve better clinical results in surgery. There were significant differences between the two groups in related surgical indexes, postoperative pain function scores and imaging findings. Although the HP technique had an advantage in surgical time (but not statistically significant), one of the outstanding advantages of the TightRope technique was that there was no need for a second surgical intervention to remove the implant, and the operation could be performed with a small incision. But this study may be subject to several limitations. First of all, this study is a single center study, which includes only patients with Rockwood type III and type IV ACJ dislocation in the same area, which has a certain regional nature and may have a certain selection bias on the accuracy of the results. In addition, the sample size of this study is relatively insufficient, which may weaken the statistical power of the final results. Finally, there may be bias in the evaluation of VAS, CMS of shoulder joint and postoperative complications. Therefore, a prospective study with a further increase in sample size or is needed to determine whether arthroscopic-assisted TightRope technique is superior to open surgery.

## Conclusion

Compared with clavicular hook plate, TightRope loop titanium button under arthroscopy is minimally invasive, safe and effective in the treatment of ACJ dislocation, and has a good clinical application trend.

## Data Availability

All data generated or analyzed during this study are included in this published article.

## Abbreviations

ACJ: Acromioclavicular joint
CCJ: Coracoclavicular joint
VAS: Visual Analogue Scale
CMS: Constant-Mueley Score

## Notes

The data used to support the findings of this study are included within the article.
